# Digital behavior therapy for insomnia in the general population: secondary analysis of the multi-arm RESiLIENT trial with active comparators

**DOI:** 10.1016/j.invent.2026.101009

**Published:** 2026-09-25

**Authors:** Masatsugu Sakata, Aran Tajika, Rie Toyomoto, Yan Luo, Tatsuo Akechi, Norito Kawakami, Masaru Horikoshi, Hisashi Noma, Toshi A. Furukawa

**Affiliations:** a Department of Neurodevelopmental Medicine, Nagoya City University Graduate School of Medical Sciences, Nagoya, Japan; b Department of Health Promotion and Behavioral Sciences, Kyoto University Graduate School of Medicine/School of Public Health, Kyoto, Japan; c International Institute for Integrative Sleep Medicine (WPI-IIIS), Tsukuba Institute for Advanced Research (TIAR), University of Tsukuba, Tsukuba, Japan; d Department of Next-Generation Organ Transplantation, The University of Tokyo Graduate School of Medicine, Tokyo, Japan; e Department of Psychiatry and Cognitive-Behavioral Medicine, Nagoya City University Graduate School of Medical Sciences, Nagoya, Japan; f Department of Digital Mental Health, Graduate School of Medicine, The University of Tokyo, Tokyo, Japan; g Department of Human Sciences, Musashino University, Tokyo, Japan; h Department of Interdisciplinary Statistical Mathematics, The Institute of Statistical Mathematics, Tokyo, Japan; i Kyoto University Office of Institutional Advancement and Communications, Kyoto, Japan

**Keywords:** Insomnia, Internet-based cognitive behavioral therapy, Behavioral activation, Digital mental health, Sleep intervention

## Abstract

**Background:**

Insomnia is common and often coexists with depression and anxiety. Digital delivery can expand access, yet few trials have compared insomnia-focused behavioral interventions with strong active controls under standardized conditions.

**Objectives:**

To examine whether a smartphone-delivered behavior therapy for insomnia (BI) improves insomnia more than a mood-focused behavioral activation (BA) module or a health-information (HI) comparator, and whether combining BI with BA (BA+BI) adds benefit.

**Methods:**

We conducted a secondary analysis of the multi-arm RESiLIENT randomized controlled trial among adults from the general population with elevated insomnia (Insomnia Severity Index (ISI) ≥8). Participants were assigned to BI, BA+BI, BA, or HI, each self-guided over six weeks. The primary outcome was ISI change at weeks 6 and 26, analyzed using mixed-effects models. Depressive (PHQ-9) and anxiety (GAD-7) symptoms were secondary outcomes. Analyses followed a modified intention-to-treat approach (≥1 post-baseline assessment).

**Results:**

At week 6, BI reduced insomnia severity more than HI (ES: -0.43, CI: −0.58 to −0.28) and BA (ES: -0.26, CI: −0.42 to −0.11); BA also exceeded HI (ES: -0.16, CI: −0.32 to −0.01). Adding BA to BI gave no benefit over BI alone (vs HI, ES: -0.30, CI: −0.45 to −0.14). BI reduced depressive (ES: -0.44, CI: −0.59 to −0.29) and anxiety symptoms (ES: -0.27, CI: −0.42 to −0.12) more than HI, and matched BA (each ES: 0.05, CI: −0.10 to 0.20). At week 26, ISI differences persisted but PHQ-9 and GAD-7 differences attenuated.

**Conclusions:**

This exploratory, hypothesis-generating analysis associated a focused smartphone-delivered sleep intervention with durable improvements in insomnia severity and mood. Replication in independent samples, and confirmation in trials recruiting participants with elevated insomnia, is required before recommendation at scale.

## Highlights

• Behavior therapy for insomnia outperformed behavioral activation and health information for insomnia at 6 and 26 weeks.
• All active modules improved depressive symptoms more than health information; gains for anxiety attenuated by 26 weeks.

## 1 Introduction

Insomnia is a common yet underrecognized public health concern, affecting up to 30% of the general population (Morin and Jarrin, 2022). It is associated with an elevated risk of subsequent mental and physical health problems. It has been identified as a strong predictor for the onset of depression, anxiety disorders (Hertenstein et al., 2019), dementia (Almondes et al., 2016), and cardiovascular disease (Sofi et al., 2014). Beyond its clinical impact, insomnia contributes substantially to population-level burden: in a large U.S. epidemiological survey, its quality-adjusted life-year (QALY) loss exceeded that of many other chronic conditions, including depression and hypertension (Olfson et al., 2018).

Cognitive behavioral therapy for insomnia (CBT—I) is the gold-standard treatment for chronic insomnia (Edinger et al., 2021; Trauer et al., 2015). Its behavioral components—particularly stimulus control and sleep restriction therapy—are supported by a strong evidence base (Furukawa et al., 2024a, Furukawa et al., 2024b; Trauer et al., 2015). However, although existing trials and meta-analyses support a substantial evidence base for CBT—I, many studies have focused comparisons with weaker control conditions such as psychoeducation, treatment as usual, or waitlist (Furukawa et al., 2024a, Furukawa et al., 2024b). Direct comparisons with strong, structurally comparable active behavioral interventions of similar credibility and intensity remain relatively limited. A small number of comparative trials have suggested that insomnia-focused interventions may have advantages over depression-focused interventions when sleep problems are prominent. For example, Blom et al., 2024, Blom et al., 2017 reported that internet-delivered CBT-I produced similar long-term improvements in depressive symptoms compared with internet-delivered CBT for depression, while showing superior effects on insomnia severity. Consequently, the specific benefits of insomnia-focused behavioral techniques—independent of nonspecific therapeutic factors such as attention or expectancy—remain unclear, particularly in general population samples.

Despite the robust evidence base, CBT-I is not readily accessible to most individuals in need because of limited therapist availability and cost barriers (Soh et al., 2020). Smartphone-based delivery provides a scalable and low-intensity alternative that can promote sleep health at the population level. Meta-analyses have demonstrated that digitally delivered CBT-I produces clinically meaningful improvements in insomnia severity, sleep quality, and related mental health outcomes, with efficacy comparable to face-to-face delivery (Gao et al., 2022; Soh et al., 2020). More recent pragmatic and smartphone-compatible interventions have also shown favorable acceptability and engagement across broader and clinically heterogeneous populations (Scott et al., 2024). Moreover, modular digital platforms can deliver distinct behavioral interventions in a standardized format, making it possible to compare their relative effects under similar conditions—an opportunity that is often difficult to achieve in traditional clinical trials.

The present study builds on the RESiLIENT trial (Resilience Enhancement with Smartphone in LIving ENvironmenTs), a large-scale, multi-arm randomized controlled trial originally designed to evaluate multiple smartphone-delivered cognitive-behavioral modules for subthreshold depression prevention and mental health promotion in the general population (Furukawa et al., 2023, Furukawa et al., 2025). The parent trial primarily examined depressive outcomes across several CBT skill modules delivered within a factorial framework. In the present exploratory secondary analysis, we specifically focused on participants with elevated insomnia symptoms to examine comparative effects on insomnia severity and related mental health outcomes among selected sleep- and mood-focused intervention arms.

Among the intervention modules, behavior therapy for insomnia (BI) incorporated stimulus control and sleep restriction therapy principles to target sleep-related behaviors directly. We also examined the effect of BI when combined with behavioral activation (BA), which was designed primarily to improve mood and increase daily activity. Because behavioral activation may promote greater daytime engagement, behavioral regularity, and mood improvement, it may also plausibly influence sleep indirectly, although it is not a primary insomnia-focused intervention.

This design enabled evaluation of two sleep-focused interventions (BI and BA+BI) against two active comparators. The health information (HI) group served as a weak but structurally matched control, appearing credible but lacking structured therapeutic content. In contrast, the BA group served as a strong active control with established antidepressant efficacy and potential secondary benefits for sleep.

This framework allowed us to test whether a sleep-focused behavioral module delivered via smartphone produces greater improvement in insomnia symptoms than either a weak or a strong active comparator, and to explore whether combining BI with BA provides any additional benefits beyond BI alone.

## 2 Methods

### 2.1 Study design

We conducted this exploratory secondary analysis based on the RESiLIENT trial, a multi-arm randomized controlled trial that evaluated the effects of internet-delivered cognitive behavioral therapy (iCBT) for depression prevention in a general adult population.

The trial enrolled over 5000 participants and delivered interventions via a purpose-built smartphone application, *Resilience-Training*, which provided structured CBT-based modules in a self-guided format. The app included five distinct modules targeting different CBT skills: behavioral activation (BA), cognitive restructuring, problem-solving, assertion training, and behavior therapy for insomnia (BI). We reported the trial protocol and primary results elsewhere (Furukawa et al., 2023, Furukawa et al., 2025). Participants were randomly assigned to parent-trial intervention arms irrespective of baseline insomnia severity or specific symptom profiles, and this secondary analysis subsequently focused on participants with elevated insomnia symptoms within the relevant randomized conditions.

We obtained ethical approval from the Ethics Committee of Kyoto University Graduate School of Medicine (C1556) and registered the trial with the UMIN Clinical Trials Registry (UMIN000047124). We conducted the original trial in accordance with the CONSORT 2010 statement (Schulz et al., 2010) and its extensions for nonpharmacological treatments (Boutron et al., 2017). We followed the same reporting principles in this secondary analysis.

For this secondary analysis, we focused on people with significant insomnia symptoms at baseline and four of the original trial arms: (1) BI, (2) BA+BI, (3) BA, and (4) health information (HI). We regarded BI and BA+BI as sleep-focused interventions and BA and HI as active comparators representing mood-focused and nonspecific conditions, respectively. Randomization, allocation concealment, and delivery procedures followed those of the parent trial. All interventions and assessments were implemented via smartphone over a six-week period.

### 2.2 Participants

Eligible participants were adults (≥18 years) of any sex with PHQ-9 scores of 14 or less, consistent with the parent trial's focus on subthreshold depressive symptoms rather than more severe depression, and currently not in treatment, recruited through an online platform. All participants provided informed consent electronically.

For this analysis, we included individuals with elevated insomnia symptoms, defined as Insomnia Severity Index (ISI) score of ≥8 at baseline (Bastien et al., 2001), a threshold often used to indicate clinically relevant insomnia complaints in community samples. We excluded participants engaged in shift work to minimize circadian disruption.

### 2.3 Intervention

Participants accessed their assigned modules through the Resilience-Training smartphone app over six weeks. Each module consisted of six self-guided chapters, with each chapter typically taking 30–40 min to complete.

The BI module specifically included daily sleep diary recording, through which participants monitored sleep patterns and applied sleep restriction and stimulus control techniques based on their self-monitored data (Watanabe et al., 2011).

The BA module delivered psychoeducation on behavioral activation using the “outside-in” principle, provided worksheets for planning and experimenting with new activities, and featured a gamified “action marathon” to maintain motivation (Furukawa et al., 2023).

In the BA + BI arm, the BA content was delivered in chapters 1–2 and the BI content in chapters 3–4, so the sleep diary was introduced in week 3 rather than week 1 (Supplementary Table 1). To support adherence, participants in BI, BA + BI, and BA groups received short, standardized encouragement messages via in-app notifications or email each week (“light human support”).

The HI group served as a structurally matched, nonspecific comparator. Participants received general health information on topics such as exercise, nutrition, and oral hygiene, presented in the same duration and interface as the active modules but without behavioral or cognitive therapy content.

For this secondary analysis, we selected the BI, BA + BI, BA, and HI arms because they allowed for the clearest comparison between insomnia-focused and depression-focused interventions, as well as between a weak structurally matched and a strong active comparator, under similar digital delivery conditions. Other approaches (e.g., cognitive restructuring, problem-solving) were excluded because they were unrelated to sleep and not pertinent to the current research question. The detailed week-by-week module structure is provided in Supplementary Table 1.

### 2.4 Baseline measures

Baseline assessments included demographic, psychosocial, and clinical variables (Table 1), consistent with the broader parent trial assessment framework (Furukawa et al., 2023). Detailed descriptions of the psychosocial measures are provided in the parent trial protocol.

**Table 1:** Baseline demographic and clinical characteristics of the cohort.

	Total	BI	BA+BI	BA	Health information
N	1005	252	244	259	250
Demographic variables					
Age (mean, SD)	43.97 (11.06)	42.44 (11.06)	43.57 (11.50)	44.99 (10.98)	44.85 (10.58)
Gender					
Male	560 (55.7%)	130 (51.6%)	144 (59.0%)	141 (54.4%)	145 (58.0%)
Female	440 (43.8%)	120 (47.6%)	100 (41.0%)	117 (45.2%)	103 (41.2%)
Others	5 (0.5%)	2 (0.8%)	0 (0.0%)	1 (0.4%)	2 (0.8%)
Marital status					
Single	242 (24.1%)	63 (25.0%)	67 (27.5%)	54 (20.9%)	58 (23.2%)
Married	701 (69.8%)	175 (69.4%)	168 (68.9%)	181 (69.9%)	177 (70.8%)
Divorced/widowed	62 (6.2%)	14 (5.6%)	9 (3.7%)	24 (9.3%)	15 (6.0%)
Number of cohabitants including self (mean, SD)	2.69 (1.32)	2.58 (1.22)	2.60 (1.33)	2.83 (1.35)	2.74 (1.36)
Education, university or higher	722 (71.8%)	177 (70.2%)	191 (78.3%)	182 (70.3%)	172 (68.8%)
Employed	911 (90.7%)	227 (90.1%)	219 (89.8%)	234 (90.3%)	231 (92.4%)
Psychosocial variables					
Big 5 personality traits (score range: 1–5) (mean, SD)					
Neuroticism	3.64 (0.79)	3.68 (0.80)	3.64 (0.82)	3.58 (0.81)	3.65 (0.74)
Extraversion	2.98 (0.92)	2.89 (0.88)	3.07 (0.89)	3.04 (0.92)	2.93 (0.97)
Openness	3.18 (0.74)	3.13 (0.76)	3.20 (0.74)	3.22 (0.74)	3.16 (0.72)
Conscientiousness	3.06 (0.78)	3.12 (0.77)	3.03 (0.78)	3.00 (0.80)	3.07 (0.78)
Agreeableness	3.18 (0.87)	3.17 (0.86)	3.27 (0.89)	3.16 (0.89)	3.11 (0.82)
Adverse Childhood Experiences (≥4 points)	90 (9.0%)	29 (11.5%)	22 (9.0%)	20 (7.7%)	19 (7.6%)
Assessment of Signal Cases (score range:0–3) (mean, SD)					
Life difficulties	0.93 (0.58)	0.89 (0.61)	0.93 (0.58)	0.93 (0.59)	0.95 (0.54)
Social support	1.29 (0.74)	1.28 (0.73)	1.31 (0.71)	1.30 (0.74)	1.29 (0.77)
Motivation	2.21 (0.40)	2.22 (0.38)	2.19 (0.42)	2.22 (0.39)	2.23 (0.39)
CBT skills (score range: 0–3) (mean, SD)					
BA skills	1.44 (0.46)	1.40 (0.41)	1.45 (0.49)	1.45 (0.46)	1.47 (0.47)
CR skills	1.37 (0.58)	1.36 (0.56)	1.35 (0.59)	1.41 (0.58)	1.34 (0.60)
PS skills	1.79 (0.56)	1.78 (0.54)	1.79 (0.59)	1.82 (0.55)	1.78 (0.56)
AT skills	1.24 (0.51)	1.19 (0.52)	1.25 (0.47)	1.26 (0.52)	1.26 (0.53)
BI skills	1.89 (0.55)	1.90 (0.55)	1.85 (0.55)	1.86 (0.55)	1.95 (0.55)
Familiarity with smartphones (score range: 0–3) (mean, SD)	1.98 (0.67)	1.96 (0.69)	1.98 (0.69)	2.01 (0.64)	1.96 (0.69)
Clinical variables					
PHQ-9 (score range: 0–27) (mean, SD)	7.72 (3.17)	7.69 (3.25)	7.78 (3.17)	7.55 (3.00)	7.86 (3.27)
GAD-7 (score range: 0–21) (mean, SD)	5.74 (3.62)	5.76 (3.68)	5.62 (3.48)	5.46 (3.50)	6.14 (3.81)
ISI (score range: 0–28) (mean, SD)	11.93 (3.45)	11.55 (3.17)	11.98 (3.50)	12.17 (3.53)	12.01 (3.57)
SWEMWBS (score range: 7–35) (mean, SD)	22.10 (3.64)	21.91 (3.68)	22.30 (3.97)	22.34 (3.43)	21.84 (3.47)
WSAS (score range: 0–8) (mean, SD)	1.82 (1.75)	1.69 (1.71)	1.88 (1.80)	1.79 (1.74)	1.92 (1.76)
EQ-5D-5L utility (score range: −0.59 to 1) (mean, SD)	0.91 (0.09)	0.91 (0.09)	0.92 (0.09)	0.91 (0.08)	0.91 (0.09)
CAGE (≥2 points)	169 (16.8%)	31 (12.3%)	40 (16.4%)	47 (18.2%)	51 (20.4%)
Past or current treatments for mental health	306 (30.5%)	82 (32.5%)	67 (27.5%)	87 (33.6%)	70 (28.0%)
Hypertension	164 (16.3%)	40 (15.9%)	44 (18.0%)	34 (13.1%)	46 (18.4%)
Diabetes mellitus	35 (3.5%)	6 (2.4%)	3 (1.2%)	14 (5.4%)	12 (4.8%)
Chronic pain	164 (16.3%)	34 (13.5%)	42 (17.2%)	43 (16.6%)	45 (18.0%)
Any physical disease	330 (32.8%)	71 (28.2%)	91 (37.3%)	80 (30.9%)	88 (35.2%)

PHQ-9: Patient Health Questionnaire-9, GAD-7: Generalized Anxiety Disorder-7, ISI: Insomnia Severity Index, WSAS: Work and Social Adjustment Scale, HPQ: Health and Work Performance Questionnaire, UWES: Utrecht Work Engagement Scale EQ-5D-5L: EuroQOL-5D-5L, SWEMWBS: Short Warwick Edinburgh Mental Well-Being Scale.

### 2.5 Outcome measures

The primary outcome was insomnia severity, assessed using the 7-item ISI, with total scores ranging from 0 to 28(Bastien et al., 2001). ISI was measured at baseline, week 3, week 6 (post-intervention), and week 26 (follow-up).

Secondary outcomes included:

• Depression symptoms, assessed weekly during the intervention (weeks 1–6) and at weeks 10, 14, 18, 22, and 26 using the Patient Health Questionnaire-9 (PHQ-9) (Kroenke et al., 2001).
• Anxiety symptoms, assessed at baseline and at weeks 3, 6, and 26 using the Generalized Anxiety Disorder-7 (GAD-7) (Spitzer et al., 2006).

We assessed adherence to the sleep diary by extracting time-stamped entries from the app. Given the self-guided nature of the intervention, adherence was pragmatically defined as recording at least one sleep diary entry within each time window to capture minimal engagement and avoid bias from excluding partial users. We summarized the number of entries per participant across four predefined time windows: weeks 0–3, 3–6, 6–26, and 26–50. Diary use was encouraged during the BI module and remained optional thereafter.

All measures were self-administered within the app. Automated reminders supported timely responses and minimized missing data.

### 2.6 Statistical analysis

We followed a modified (adapted) intention-to-treat principle: all randomized participants who met the ISI ≥8 criterion and provided at least one post-baseline outcome assessment were included, in the group to which they were randomized. Of the 1005 participants, 987 did so and entered the models (BI 249 of 252, BA+BI 237 of 244, BA 253 of 259, HI 248 of 250); the remaining 18 provided no post-baseline data and could not contribute to any model. Because this was a secondary and exploratory analysis, statistical power was determined by the primary analysis, which was designed to detect differences in depressive symptoms (PHQ-9). Analysis of insomnia and anxiety outcomes was therefore descriptive hypothesis-generating.

We used mixed-effects models for repeated measures (MMRM) with unstructured covariance to analyze all outcomes, allowing flexible modeling of within-participant correlations over time while minimizing assumptions about the covariance structure. Under the missing-at-random assumption, MMRM used all available repeated observations from participants with at least one post-baseline assessment without formal imputation. Each model included fixed effects for time, treatment group, time-by-treatment interaction, and baseline outcome values.

To improve estimate precision at weeks 6 and 26, we incorporated all available repeated data for each outcome (baseline, weeks 3, 6 and 26 for ISI and GAD-7, which were administered at those visits only; baseline, weeks 1–6, 10, 14, 18, 22 and 26 for PHQ-9).

We reported marginal means, standardized mean differences (SMDs) divided by the observed week 6 SD, 95% confidence intervals, and *p*-values derived from model-based standard errors. We performed all analyses using R version 4.3.2 and the *mmrm* package.

## 3 Results

### 3.1 Participant flow and baseline characteristics

This secondary analysis included 1005 participants with elevated insomnia symptoms (ISI ≥ 8) at baseline, drawn from a larger randomized trial. Participants were allocated to one of four groups: behavioral intervention (BI, *n* = 252), behavioral activation (BA, *n* = 259), combined BA+BI (*n* = 244), or health information control (HI, *n* = 250).

Retention was 97.3% at week 6 and 91.4% at week 26 (Fig. 1). Baseline demographic and clinical characteristics (age, gender, ISI, PHQ-9, and GAD-7) were well-balanced across groups (Table 1).

**Fig. 1:**
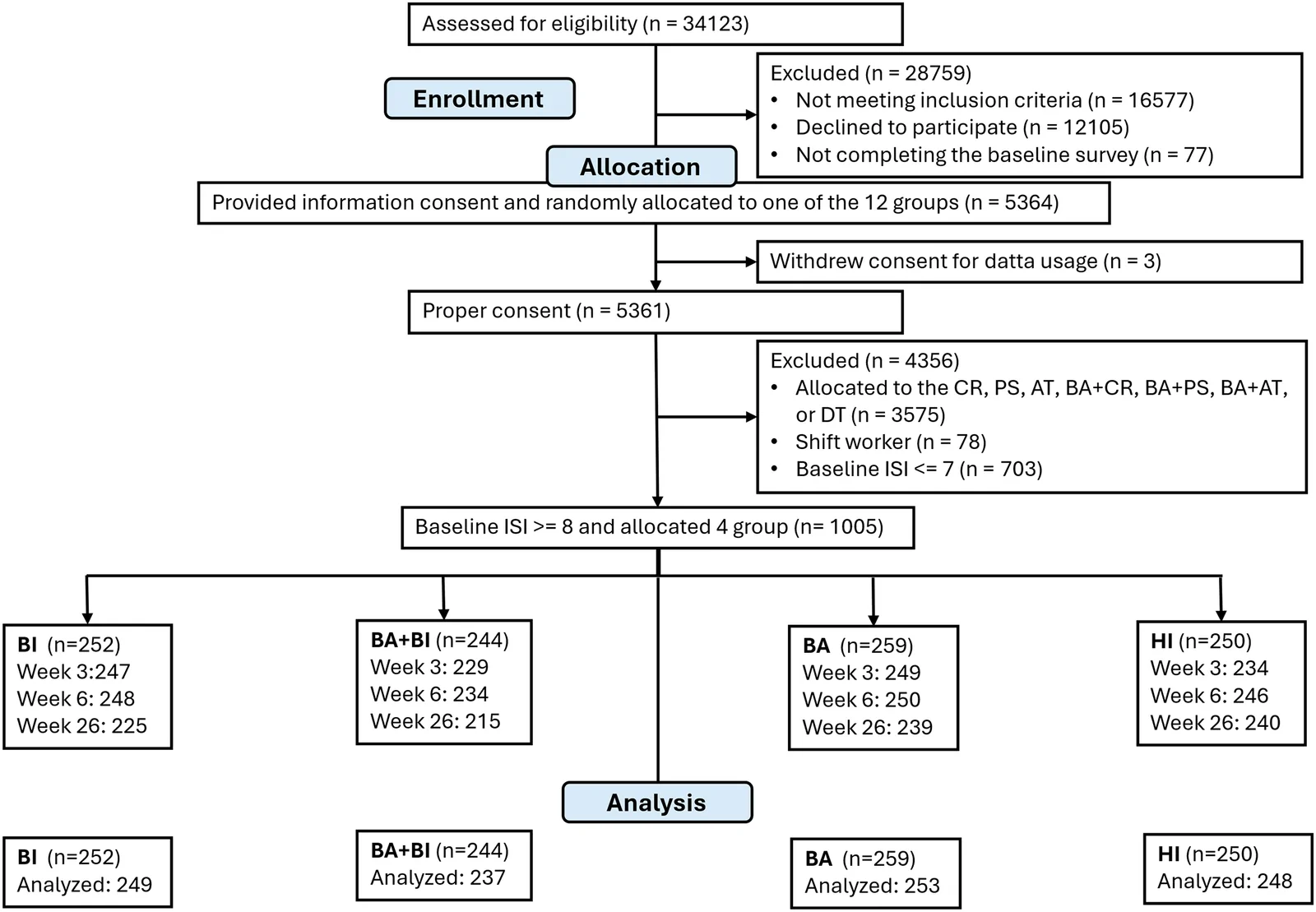
CONSORT flowchart (baseline ISI ≥ 8). AT: assertion training, BA: behavioral activation, BI: behavior therapy for insomnia, CR: cognitive restructuring, ISI: Insomnia Severity Index, PS: problem solving.

### 3.2 Primary outcome: Insomnia Severity Index (ISI)

Insomnia symptoms decreased from baseline in all groups at both week 6 and week 26 (Table 2, Fig. 2). At week 6, BI showed greater reductions in insomnia severity than BA (effect size = −0.26, 95% CI −0.42 to −0.11) and HI (effect size = −0.43, 95% CI −0.58 to −0.28). Similar between-group differences were maintained at week 26, with BI outperforming BA (effect size = −0.25, 95% CI −0.43 to −0.08) and HI (effect size = −0.44, 95% CI −0.61 to −0.27). BA+BI also showed greater improvement than HI but did not clearly exceed BI alone. Overall, the results indicate that the smartphone-delivered sleep-focused intervention (BI) achieved the most sustained reduction in insomnia symptoms among adults with significant insomnia in the general population.

**Table 2:** Changes in ISI scores from baseline to week 6 and to week 26 using the Mixed-Effects Model for Repeated Measures (MMRM) (baseline ISI ≥ 8).

2a. Week 6								
Arm	N	N (Week 6)	Estimated least squares mean baseline-Week 6 change scores^a^ (95% CI)	Effect size for baseline-week 6 change scores^b^ (95% CI)	Estimated differences of change scores vs HI^b^		Effect size for differences of change scores (95% CI)	
					(95% CI)	*P*-value	vs HI	vs BA
BI	252	248	−3.61 (−4.37 to −2.85)	−1.05 (−1.27 to −0.83)	−1.93 (−2.62 to −1.24)	<0.001	−0.43 (−0.58 to −0.28)	−0.26 (−0.42 to −0.11)
BA + BI	244	234	−3.01 (−3.79 to −2.24)	−0.87 (−1.10 to −0.65)	−1.33 (−2.03 to −0.63)	<0.001	−0.30 (−0.45 to −0.14)	−0.13 (−0.29 to 0.02)
BA	259	250	−2.41 (−3.18 to −1.66)	−0.70 (−0.92 to −0.48)	−0.76 (−1.42 to −0.05)	0.035	−0.16 (−0.32 to −0.01)	–
Health information [reference]	250	246	−1.68 (−2.44 to −0.92)	−0.49 (−0.71 to −0.27)	–	–	–	–


2b. Week 26								
Arm	N	N (Week 26)	Estimated least squares mean baseline-Week 26 change scores^a^ (95% CI)	Effect size for baseline-week 26 change scores^b^ (95% CI)	Estimated differences of change scores vs HI^b^		Effect size for differences of change scores (95% CI)	
					(95% CI)	*P*-value	vs HI	vs BA
BI	252	225	−3.50 (−4.31 to −2.69)	−1.01 (−1.25 to −0.78)	−1.98 (−2.77 to −1.19)	<0.001	−0.44 (−0.61 to −0.27)	−0.25 (−0.43 to −0.08)
BA + BI	244	215	−3.11 (−3.94 to −2.28)	−0.90 (−1.14 to −0.66)	−1.59 (−2.39 to −0.80)	<0.001	−0.35 (−0.53 to −0.18)	−0.17 (−0.34 to 0.01)
BA	259	239	−2.37 (−3.17 to −1.56)	−0.69 (−0.92 to −0.45)	−0.85 (−1.62 to −0.07)	0.033	−0.19 (−0.36 to −0.02)	–
Health information [reference]	250	240	−1.51 (−2.32 to −0.72)	−0.44 (−0.67 to −0.21)	–	–	–	–

BA: behavioral activation, BI: behavior therapy for insomnia, ISI: Insomnia Severity Index.

a Minus value indicates insomnia reduction. () indicates 95% confidence intervals.

b Effect sizes (standardized mean differences) were calculated using the observed baseline SD (3.45) for within-group change scores and the observed week 6 SD (4.51) for group differences.

**Fig. 2:**
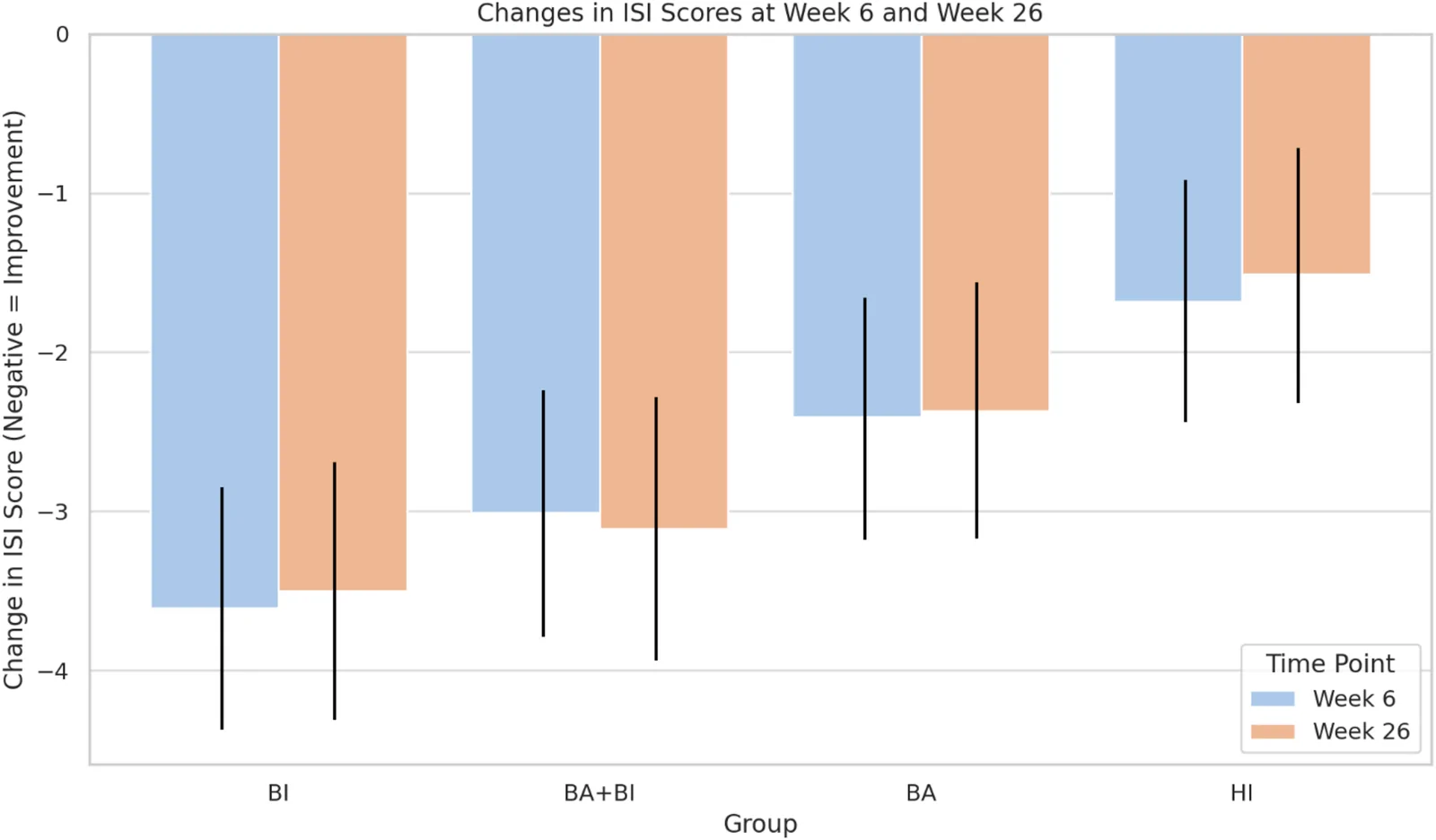
Estimated mean changes in ISI scores from baseline to week 6 and week 26 by intervention group, based on the mixed-effects model for repeated measures (MMRM). Error bars represent 95% confidence intervals. Negative values indicate improvements in insomnia severity. BA: Behavioral activation, BI: Behavior therapy for insomnia, ISI: Insomnia Severity Index.

### 3.3 Secondary outcomes: PHQ-9 and GAD-7

For depressive symptoms (PHQ-9), all active modules (BI, BA+BI, and BA) produced greater reductions than HI at both post-intervention and follow-up (Table 3). Differences among the active interventions were comparatively small, with BI and BA showing broadly similar antidepressant effects over time. For anxiety (GAD-7), BI and BA participants improved more than HI at week 6, but these between-group differences were smaller and less consistently maintained at week 26 (Table 4). These findings suggest that digital behavioral interventions targeting either sleep or mood can concurrently alleviate anxiety and depression symptoms, although the persistence of effects may differ by domain.

**Table 3:** Changes in PHQ-9 scores from baseline to week 6 and to week 26 using the Mixed-Effects Model for Repeated Measures (MMRM) (baseline ISI ≥ 8).

3a. Week 6								
Arm	N	N (Week 6)	Estimated least squares mean baseline-Week 6 change scores^a^ (95% CI)	Effect size for baseline-week 6 change scores^b^ (95% CI)	Estimated differences of change scores vs HI^b^		Effect size for differences of change scores (95% CI)	
					(95% CI)	*P*-value	vs HI	vs BA
BI	252	248	−3.08 (−3.67 to −2.50)	−0.82 (−0.98 to −0.67)	−1.66 (−2.22 to −1.09)	<0.001	−0.44 (−0.59 to −0.29)	0.05 (−0.10 to 0.20)
BA + BI	244	234	−2.79 (−3.38 to −2.19)	−0.74 (−0.90 to −0.58)	−1.36 (−1.93 to −0.79)	<0.001	−0.36 (−0.52 to −0.21)	0.13 (−0.03 to 0.28)
BA	259	250	−3.26 (−3.84 to −2.67)	−0.87 (−1.02 to −0.71)	−1.83 (−2.40 to −1.27)	<0.001	−0.49 (−0.64 to −0.34)	–
Health information [reference]	250	246	−1.43 (−2.01 to −0.84)	−0.38 (−0.54 to −0.22)	–	–	–	–


3b. Week 26								
Arm	N	N (Week 26)	Estimated least squares mean baseline-Week 26 change scores^a^ (95% CI)	Effect size for baseline-week 26 change scores^b^ (95% CI)	Estimated differences of change scores vs HI^b^		Effect size for differences of change scores (95% CI)	
					(95% CI)	*P*-value	vs HI	vs BA
BI	252	225	−2.69 (−3.34 to −2.05)	−0.72 (−0.89 to −0.55)	−0.97 (−1.65 to −0.30)	0.005	−0.26 (−0.44 to −0.08)	0.03 (−0.15 to 0.21)
BA + BI	244	215	−2.76 (−3.41 to −2.10)	−0.73 (−0.91 to −0.56)	−1.04 (−1.72 to −0.36)	0.003	−0.28 (−0.46 to −0.10)	0.01 (−0.17 to 0.20)
BA	259	239	−2.81 (−3.44 to −2.17)	−0.75 (−0.92 to −0.58)	−1.09 (−1.76 to −0.42)	0.001	−0.29 (−0.47 to −0.11)	
Health information [reference]	250	240	−1.72 (−2.35 to −1.08)	−0.46 (−0.63 to −0.29)	–	–	–	–

BA: behavioral activation, BI: behavior therapy for insomnia, ISI: Insomnia Severity Index, PHQ-9: Patient Health Questionnaire-9.

a Minus value indicates insomnia reduction. () indicates 95% confidence intervals.

b Effect sizes (standardized mean differences) were calculated using the observed week 6 SD (3.75).

**Table 4:** Changes in GAD-7 scores from baseline to week 6 and to week 26 using the Mixed-Effects Model for Repeated Measures (MMRM) (baseline ISI ≥ 8).

4a. Week 6								
Arm	N	N (Week 6)	Estimated least squares mean baseline-Week 6 change scores^a^ (95% CI)	Effect size for baseline-week 6 change scores^b^ (95% CI)	Estimated differences of change scores vs HI^b^		Effect size for differences of change scores (95% CI)	
					(95% CI)	*P*-value	vs HI	vs BA
BI	252	248	−2.12 (−2.70 to −1.54)	−0.62 (−0.79 to −0.45)	−0.91 (−1.42 to −0.40)	<0.001	−0.27 (−0.42 to −0.12)	0.05 (−0.10 to 0.20)
BA + BI	244	234	−1.67 (−2.26 to −1.08)	−0.49 (−0.67 to −0.32)	−0.46 (−0.97 to 0.05)	0.078	−0.14 (−0.29 to 0.02)	0.18 (0.03 to 0.33)
BA	259	250	−2.29 (−2.86 to −1.71)	−0.67 (−0.84 to −0.50)	−1.08 (−1.58 to −0.57)	<0.001	−0.32 (−0.47 to −0.17)	–
Health information [reference]	250	246	−1.21 (−1.79 to −0.63)	−0.36 (−0.53 to −0.19)	–	–	–	–


4b. Week 26								
Arm	N	N (Week 26)	Estimated least squares mean baseline-Week 26 change scores^a^ (95% CI)	Effect size for baseline-week 26 change scores^b^ (95% CI)	Estimated differences of change scores vs HI^b^		Effect size for differences of change scores (95% CI)	
					(95% CI)	*P*-value	vs HI	vs BA
BI	252	225	−1.85 (−2.47 to −1.22)	−0.54 (−0.73 to −0.36)	−0.44 (−1.04 to 0.16)	0.152	−0.13 (−0.31 to 0.05)	0.04 (−0.14 to 0.21)
BA + BI	244	215	−1.82 (−2.46 to −1.19)	−0.54 (−0.72 to −0.35)	−0.42 (−1.02 to 0.19)	0.180	−0.13 (−0.30 to 0.06)	0.04 (−0.14 to 0.22)
BA	259	239	−1.96 (−2.58 to −1.34)	−0.58 (−0.76 to −0.40)	−0.56 (−1.15 to 0.04)	0.064	−0.16 (−0.34 to 0.01)	–
Health information [reference]	250	240	−1.41 (−2.02 to −0.79)	−0.41 (−0.60 to −0.23)	–	–	–	–

BA: behavioral activation, BI: behavior therapy for insomnia, GAD-7: Generalized Anxiety Disorder-7, ISI: Insomnia Severity Index.

a Minus value indicates insomnia reduction. () indicates 95% confidence intervals.

b Effect sizes (standardized mean differences) were calculated using the observed week 6 SD (3.40).

### 3.4 Sleep diary adherence

Adherence to the sleep diary was moderate overall (Table 5). Participants in the BI group completed more diary entries than those in the BA+BI group during the intervention period, and this pattern persisted throughout the follow-up period. Higher diary engagement in BI may reflect the simplicity and focus of the single-module design.

**Table 5:** Sleep diary adherence across intervention and follow-up periods.

Arm	N		Weeks 0–3	Weeks 3–6	Weeks 6–26	Weeks 26–50
BI	252	n with ≥1 sleep diary entry	247	246	230	157
		Mean number of sleep diary entries (SD)	18.19 (3.78)	18.35 (4.19)	78.70 (55.41)	92.39 (63.26)
BA+BI	244	n with ≥1 sleep diary entry	–	216	205	108
		Mean number of sleep diary entries (SD)	–	16.44 (5.07)	56.02 (52.66)	77.56 (68.93)

BA: behavioral activation, BI: behavior therapy for insomnia.

## 4 Discussion

### 4.1 Key findings

This exploratory secondary analysis found that an insomnia-focused behavioral module (BI) delivered via smartphone produced the greatest and most sustained reductions in insomnia symptoms among adults from the general population with elevated insomnia severity. BI showed larger improvements in insomnia than both the mood-focused behavioral activation (BA) module and the health-information (HI) comparator, while achieving comparable improvements in depressive and anxiety symptoms. These results indicate that although both BI and BA have transdiagnostic benefits, a focused insomnia intervention may be the preferred first-line approach when sleep problems are prominent.

Adding BA to BI did not enhance outcomes on insomnia, depression, or anxiety measures. Although BI and BA both target behavior change, they differ in focus—BI addresses sleep-related habits and conditioning processes, whereas BA seeks to increase daily reward and activity. However, because the BI content was introduced later in the BA + BI arm (week 3 rather than week 1), the absence of additional benefit may partly reflect the shorter period of sleep-diary practice rather than an inherent disadvantage of combined behavioral approaches.

The pattern of improvements in depressive and anxiety symptoms was similar across active interventions, supporting previous findings that behavioral approaches can produce transdiagnostic benefits. Adherence data suggested that participants maintained self-monitoring behaviors beyond the intervention period, particularly in the BI group, indicating that a concise digital insomnia module can foster sustainable behavioral routines. The similar antidepressant effects observed across BI, BA, and BA+BI despite differing primary intervention targets may reflect overlapping mechanisms such as improved behavioral regularity, daytime functioning, or mood–sleep interdependence, through which both sleep-focused and mood-focused interventions may produce broader psychological benefits.

### 4.2 Comparison with existing research

Our results extend prior evidence on digital interventions for insomnia by directly comparing an insomnia-focused behavioral module with both a mood-focused behavioral module and a nonspecific informational control, all delivered within a unified digital platform under standardized conditions. A recent meta-analysis (Furukawa et al., 2024a, Furukawa et al., 2024b) identified more than 60 randomized trials of behavior therapy for insomnia, typically incorporating behavioral techniques such as stimulus control and sleep restriction without cognitive components. However, only a small fraction of those trials included more than two arms or employed active behavioral comparators. Consistent with this gap, Blom et al., 2017, Blom et al., 2024 directly compared internet-delivered CBT for insomnia with internet-delivered CBT for depression among patients with both insomnia and depression, finding similar improvements in depressive symptoms but superior long-term effects on insomnia severity for the insomnia-focused intervention. The present study therefore contributes to this limited evidence base by evaluating an insomnia-focused intervention against both weak and strong active comparators in a multi-arm digital trial.

Previous digital CBT-I studies, including commercial and regulatory-approved programs (Soh et al., 2020), have demonstrated efficacy compared with non-active controls. However, few trials have examined the relative contribution of different behavioral approaches within the same digital environment. By embedding multiple behavioral modules into a single platform, the RESiLIENT trial enabled a direct comparison of insomnia- and mood-focused interventions with equivalent intensity and structure. Our findings showed that the targeted BI module provided more durable improvement in insomnia than broader mood-focused content, highlighting the value of focused, behaviorally specific design in digital health applications.

### 4.3 Public health implications

From a public-health perspective, these results support the use of brief, scalable, and insomnia-focused digital interventions to address widespread insomnia symptoms in the community. Because BI alone was effective and well-engaged, it represents a low intensity yet impactful option for improving population sleep health at scale.

Importantly, BI also yielded improvements in mood and anxiety, indicating potential transdiagnostic and preventive utility. Early, accessible interventions that target insomnia may help mitigate downstream mental-health problems, as insomnia is a known precursor of depression and anxiety. Such programs could therefore function as preventive public-health tools, complementing higher-intensity treatments for clinical insomnia within stepped-care models or national digital health platforms.

In sum, provided these exploratory findings are confirmed in a purpose-designed trial, a simple, smartphone-delivered BI module may serve dual purposes: as a scalable population-level intervention to improve insomnia and wellbeing, and as a low-intensity entry point within clinical care pathways for individuals with emerging sleep complaints.

### 4.4 Strengths and limitations

A major strength of this study is its ability to estimate the specific effect of insomnia-focused behavioral intervention while accounting for nonspecific treatment factors. By including both a weak active control (HI) and a strong behavioral comparator (BA), the design allowed a more precise attribution of improvements in insomnia symptoms to the behavioral intervention itself. The standardized smartphone-based platform also enabled uniform delivery of all modules and direct comparison of distinct behavioral approaches under identical digital conditions—an approach that would be difficult to achieve in face-to-face trials.

In addition, the large sample size and high retention rates enhance the statistical precision, robustness, and longitudinal stability of the findings. Although this secondary analysis was exploratory and the parent trial was originally powered for depressive outcomes, the consistency and durability of effects across time points suggest that the observed patterns are robust.

These findings should be read as exploratory and hypothesis-generating rather than confirmatory. The parent trial was designed and powered to detect differences in depressive symptoms among community adults with subthreshold depression; participants were not recruited on the basis of sleep complaints, insomnia disorder was not diagnostically ascertained, and the ISI ≥8 subgroup and the four-arm comparisons reported here were defined for this secondary analysis rather than pre-specified in the parent protocol. No adjustment for multiplicity was applied across the outcomes and contrasts examined. Consequently, the estimates reported here generate hypotheses about the comparative effects of sleep- versus mood-focused digital modules; they do not test them. They require replication in independent samples, and confirmation in a trial designed and powered a priori for this question, recruiting participants selected for elevated insomnia symptoms—and, where the transdiagnostic claim is of interest, for elevated depressive symptoms as well.

Several limitations should be acknowledged. First, because participants were recruited from the general population, the findings may not fully generalize to patients with clinically diagnosed insomnia or comorbid conditions. Moreover, because participants were selected based on elevated insomnia symptoms (ISI ≥8) rather than formal diagnostic criteria for insomnia disorder, this sample likely reflects a broader population with clinically relevant sleep complaints rather than strictly diagnosed insomnia disorder. This approach may enhance public-health relevance but may limit direct generalizability to narrowly defined clinical insomnia populations. Second, the sequential delivery order in the BA + BI arm limited exposure to BI during the first half of the intervention, which may have reduced its relative impact at the post-treatment stage. Third, because a modified intention-to-treat approach was used, participants who provided no post-baseline data could not contribute to the models; estimates rest on a missing-at-random assumption that cannot be verified. Finally, the study relied on self-guided engagement without therapist involvement; while this enhances scalability, it may limit personalization and support for users requiring higher-intensity care. Future trials should examine strategies to tailor digital insomnia interventions to individual needs and integrate them effectively within stepped-care or public health implementation frameworks.

### 4.5 Future directions

Future research should determine the minimal effective dose of digital insomnia interventions and evaluate adaptive delivery formats tailored to individual symptom patterns. Hybrid models that combine self-guided modules with therapist- or AI-based support could further optimize adherence and scalability. Longitudinal implementation studies within healthcare systems and population settings will clarify how digital insomnia interventions can contribute to public-health prevention frameworks.

## 5 Conclusion

In this exploratory secondary analysis of a general-population trial, smartphone-delivered behavior therapy for insomnia (BI) was associated with the greatest and most durable improvements in insomnia severity among the digital modules compared, and adding behavioral activation did not enhance these effects. Because the analysis was hypothesis-generating rather than confirmatory—the subgroup and comparisons were not pre-specified, and the trial was powered for depressive rather than insomnia outcomes—these results should not be taken as establishing the superiority of BI. They indicate that focused, simple and scalable sleep interventions warrant direct evaluation, and require replication in independent samples and confirmation in trials designed a priori to test these comparisons among participants recruited for elevated insomnia (and depressive) symptoms.

## Abbreviations

BI: Behavior therapy for insomnia
BA: Behavioral activation
BA+BI Combined: Behavioral activation and behavior therapy for insomnia
HI: Health information
CBT-I: Cognitive behavioral therapy for insomnia
RCT: Randomized controlled trial
RESiLIENT: Resilience Enhancement with Smartphone in LIving ENvironmenTs (trial name)
ISI: Insomnia Severity Index
PHQ-9: Patient Health Questionnaire-9
GAD-7: Generalized Anxiety Disorder 7-item scale
SMD: Standardized mean difference
QALY: Quality-adjusted life year
TAU: Treatment as usual
JCSM: Journal of Clinical Sleep Medicine
AMED: Japan Agency for Medical Research and Development
UMIN-ICDR: University Hospital Medical Information Network – Individual Case Data Repository

## CRediT authorship contribution statement

T.A.F. and M.H. conceived the study. T.A.F. and H.N. designed the study. Funding was obtained by T.A.F., M.H., T.A., N. Kawakami. T.A.F., M.H. and T.A. led the software development, with input from A.T., R.T., M.S. and Y.L. A.T., R.T., M.S. and Y.L. established the databases and managed the recruitment, data collection and processing, with help from N. Kawakami. M.S. and T.A.F. undertook the statistical analyses under supervision of H. N., and had direct access to the full data. M.S. and T.A.F. wrote the first draft, and all the authors contributed to the interpretation and critical revision of the manuscript, and approved the final paper.

## Ethics approval

This study was approved by the ethics committee of Kyoto University Graduate School of Medicine (Approval No. C1556) on 3 March 2022, and informed consent was obtained from all participants online.

## Author approval statement

All authors have reviewed and approved the final version of the manuscript.

## Registration information

UMIN000047124.

## Declaration of Generative AI and AI-assisted technologies in the writing process

We used generative AI assistants (ChatGPT, OpenAI; Claude, Anthropic) to support language polishing, formatting, and consistency checks during manuscript preparation. We (the authors) critically reviewed and edited all AI-assisted text, verified the accuracy and integrity of the scientific content, and take full responsibility for the manuscript. The AI systems did not generate, analyze, or interpret data, and we did not input any identifiable participant information into the tool. Use of AI complied with journal policies and institutional guidance.

## Funding sources

This study was supported by the 10.13039/100009619Japan Agency for Medical Research and Development (AMED) (grant no. JP21de0107005), awarded to Toshi A. Furukawa (principal investigator), Aran Tajika, Rie Toyomoto, Masatsugu Sakata, Yan Luo, Masaru Horikoshi., Tatsuo Akechi, Norito Kawakami, and Hisashi Noma. The funder had no role in study design, data collection, data analysis, data interpretation, or report writing.

## Declaration of competing interest

Masatsugu Sakata is employed in the Department of Neurodevelopmental Disorders, Nagoya City University Graduate School of Medical Sciences, which is an endowment department supported by the City of Nagoya, and has received a personal fee from SONY outside the submitted work.

Toshi A. Furukawa reports personal fees from Boehringer Ingelheim, Daiichi Sankyo, DT Axis, Micron, Shionogi, SONY and UpToDate, and a grant from DT Axis and Shionogi, outside the submitted work. In addition, Toshi. A. Furukawa has a patent (7448125) and a pending patent (2022-082495) and has licensed intellectual properties for Kokoro-app to DT Axis.

Aran Tajika reports personal fees from Shionogi and Otsuka outside the submitted work.

Masaru Horikoshi has a patent (7448125) and has licensed intellectual properties for Kokoro-app to Mitsubishi Tanabe.

Tatsuo Akechi has received lecture fees from AstraZeneca, Chugai, Daiichi Sankyo, Eisai, Eli Lilly, Kowa, Kyowa Kirin, Lundbeck, MSD, Meiji-Seika Pharma, Merck, Nipro, Otsuka, Pfizer, Shionogi, Sumitomo pharma, Takeda, Tsumura, UCB and Viatris, and a grant from Shinogi and royalties from Igaku-Shoin, outside the submitted work. Tatsuo Akechi serves as the representative director of the General Incorporated Association NCU CRESS and receives compensation as an advisor to Snom Inc. Tastuo Akechi has pending patents (2020-135195 and 2024-516288) (Institute) and patents (7313617) (Institute).

Norito Kawakami is employed by the Junpukai Foundation and the Department of Digital Mental Health, The University of Tokyo, an endowment department that is supported by an unrestricted grant from 15 enterprises (https://dmh.m.u-tokyo. ac.jp/en).

Hisashi Noma received a research fund from GlaxoSmithKline and consulting fees from GlaxoSmithKline, Sony, and Kowa, outside the submitted work.

## Data Availability

Deidentified individual participant data and data dictionary will be made available 24 months after publication on UMIN-ICDR, an individual case data repository managed by the Japanese University Hospital Medical Information Network (UMIN) Center (https://www.umin.ac.jp/icdr/index.html).
