# Supplementary material for: Digital behavior therapy for insomnia in the general population: secondary analysis of the multi-arm RESiLIENT trial with active comparators
Source: Internet Interv. 2026 Sep 25;46:101009. doi: 10.1016/j.invent.2026.101009 (PMC13671329; doi:10.1016/j.invent.2026.101009)
Supplement: Supplementary file 1 [file mmc1.docx]

# SUPPLEMENTAL MATERIAL

## Digital behavior therapy for insomnia in the general population: secondary analysis of the multi-arm RESiLIENT trial with active comparators

**Supplementary Table 1 Proceedings of the intervention and control arms**

|  | Introduction | Chapter 1 | Chapter 2 | Chapter 3 | Chapter 4 | Chapter 5 | Chapter 6 |
| --- | --- | --- | --- | --- | --- | --- | --- |
| BA | 3 lessons for psychoeducation | 4 basic lessons for BA | 2 advanced lessons for BA | 1 advanced lesson for BA | 1 advanced lesson for BA | BA in everyday life | Epilogue |
| BI | 3 lessons for psychoeducation | 5 basic lessons for BI | 1 advanced lesson for BI | 1 advanced lesson for BI | 1 advanced lesson for BI | BI in everyday life | Epilogue |
| BA+BI | 3 lessons for psychoeducation | 4 basic lessons for BA | 4 advanced lessons for BA | 5 basic lessons for BI | 3 advanced lessons for BI | BA&BI in everyday life | Epilogue |
| Health information | 1 lesson for psychoeducation | Physical exercise | Nutrition | Oral health | - | - | Epilogue |

**Supplementary Table 2 Observed ISI scores through the trial**

|  | Week 0 |  |  |  | Week 3 |  |  |  | Week 6 |  |  |  | Week 26 |  |  |  |
| --- | --- | --- | --- | --- | --- | --- | --- | --- | --- | --- | --- | --- | --- | --- | --- | --- |
| Arm | N | Mean |  | SD | N | Mean |  | SD | N | Mean |  | SD | N | Mean |  | SD |
| BI | 252 | 11.55 |  | 3.17 | 247 | 8.45 |  | 3.73 | 248 | 7.76 |  | 3.96 | 225 | 7.78 |  | 3.69 |
| BA+BI | 244 | 11.98 |  | 3.50 | 229 | 9.96 |  | 4.19 | 234 | 8.60 |  | 4.33 | 215 | 8.53 |  | 4.51 |
| BA | 259 | 12.17 |  | 3.53 | 249 | 10.44 |  | 4.28 | 250 | 9.32 |  | 4.77 | 239 | 9.45 |  | 5.22 |
| Health information | 250 | 12.01 |  | 3.57 | 234 | 10.53 |  | 4.27 | 246 | 10.03 |  | 4.63 | 240 | 10.24 |  | 5.24 |
| Follow-up proportion | 100% |  |  |  | 95.4% |  |  |  | 97.3% |  |  |  | 91.4% |  |  |  |

BA: Behavioral activation, BI: Behavior therapy for insomnia.
